# Optimizing Soil Health and Maize Yield Under Salinity Stress with Compost and Sulfur Nanoparticles

**DOI:** 10.3390/plants14111661

**Published:** 2025-05-29

**Authors:** Mahmoud M. A. Shabana, Nevien Elhawat, Mohamed A. Abd El-Aziz, S. H. Abd Elghany, Asmaa F. M. Badawy, Tarek Alshaal

**Affiliations:** 1 Soils, Water and Environment Research Institute (SWERI), Agricultural Research Center, Giza 12619, Egypt; shabanamma@gmail.com (M.M.A.S.); m.abdelaziz6275@gmail.com (M.A.A.E.-A.); haassaann72@gmail.com (S.H.A.E.); asmaafathy404@yahoo.com (A.F.M.B.); 2 Institute of Applied Plant Biology, Faculty of Agricultural and Food Sciences and Environmental Management, University of Debrecen, 4032 Debrecen, Hungary; 3 Faculty of Agriculture (for Girls), Al-Azhar University, Nasr City, Cairo 11884, Egypt; 4 Soil and Water Science Department, Faculty of Agriculture, Kafrelsheikh University, Kafr El-Sheikh 33516, Egypt

**Keywords:** soil salinity mitigation, compost–sulfur synergy, maize growth enhancement, soil health improvement, saline soil reclamation, nutrient availability optimization

## Abstract

Soil salinity poses a significant challenge to agricultural productivity, particularly in arid and semi-arid regions. This study explores the effect of compost, elemental sulfur (ES), sulfur nanoparticles (SNPs), and their combinations, i.e., compost + ES and compost + SNPs, to improve soil properties and maize productivity across a range of salinity levels (EC1 = 3.68, EC2 = 6.15, EC3 = 8.34, and EC4 = 12.18 dS/m). We hypothesized that integrating compost with ES or SNPs would enhance soil quality and maize performance more effectively than individual treatments. Results validated this hypothesis, showing that compost increased soil organic matter (SOM) by 1.33 times, reduced sodium adsorption ratio (SAR) by 33%, and boosted maize grain yield by 40% in moderately saline soils (6.15 dS/m). ES and SNPs lowered soil pH by 0.8–1.2 units and improved phosphorus availability by 25–30%. The compost–SNP combination delivered the most significant improvements, enhancing infiltration rate by 60%, total porosity by 15%, and straw yield by 50% in highly saline soils (12.18 dS/m). Additionally, plant height, cob length, and chlorophyll content increased by 20%, 22%, and 40%, respectively, with combined treatments. These findings highlight the efficacy of integrated amendments in alleviating salinity stress, offering a promising strategy for sustainable agriculture in saline environments.

## 1. Introduction

Soil salinity poses a significant obstacle to global agricultural productivity. It impacts approximately 20% of the world’s irrigated lands and 7% of drylands. This issue is worsening due to unsustainable irrigation practices. Climate change also contributes to the problem. Additionally, rising groundwater tables further exacerbate soil salinity. Saline soils, characterized by high concentrations of soluble salts, adversely affect soil structure, nutrient availability, and plant growth, leading to reduced crop yields and economic losses [1]. The problem is exacerbated by climate change, improper irrigation practices, and the use of saline groundwater, which further increase soil salinity levels [2]. Maize (*Zea mays* L.), a staple crop widely cultivated for food, feed, and industrial purposes, is particularly sensitive to salinity stress, which inhibits its growth, nutrient uptake, and overall productivity [3]. Therefore, developing effective strategies to mitigate salinity stress and improve soil health is essential for sustainable agriculture.

Soil amendments, such as compost, elemental sulfur (ES), and sulfur nanoparticles (SNPs), have gained attention for their potential to alleviate salinity stress and enhance soil properties. Compost, a rich source of organic matter, improves soil structure, water retention, and nutrient availability, thereby promoting plant growth under saline conditions [4]. Sulfur-based amendments, including ES and SNPs, play a crucial role in soil reclamation by lowering soil pH, improving nutrient solubility, and reducing the toxic effects of sodium (Na^+^) and chloride (Cl^−^) ions [5]. SNPs, in particular, have shown promise due to their high surface area and reactivity, which enhance their efficiency in soil remediation and plant stress alleviation [6].

Several studies have demonstrated the effectiveness of soil amendments in mitigating salinity stress and improving crop productivity. For instance, the application of compost significantly improved soil organic carbon, nutrient availability, and maize yield under saline conditions [5]. Similarly, sulfur amendments were found to reduce soil pH and enhance the availability of essential nutrients, such as phosphorus (P) and potassium (K), in saline soils [7]. SNPs, due to their unique properties, have been reported to improve soil microbial activity, nutrient cycling, and plant growth under stress conditions [8]. Combining compost with ES or SNPs has also shown synergistic effects, further enhancing soil properties and crop performance [9].

Despite the promising results, the effectiveness of soil amendments in alleviating salinity stress can vary depending on soil type, salinity level, and amendment composition. For instance, the response of maize to compost and S amendments was found to be more pronounced in moderately saline soils compared to highly saline soils [10]. Additionally, the interaction between soil amendments and soil microbial communities plays a crucial role in determining their effectiveness. While this study did not directly measure microbial activity, the improvements in soil organic matter and nutrient availability suggest that the amendments may enhance microbial diversity and activity, which are critical for nutrient cycling and overall soil health. Studies have shown that compost and SNP amendments enhance microbial diversity and activity, which in turn improve nutrient cycling and plant stress tolerance [11]. Therefore, a comprehensive understanding of the interactions between soil amendments, soil properties, and plant responses is essential for optimizing their use in saline soil reclamation

The choice of salinity levels (e.g., (EC1 = 3.68, EC2 = 6.15, EC3 = 8.34, and EC4 = 12.18 dS/m)) in this study is based on the need to evaluate the effectiveness of soil amendments across a range of conditions, from moderate to high salinity. Moderately saline soils (EC1) are common in many agricultural regions, while highly saline soils (EC4) represent more extreme conditions that are increasingly prevalent due to climate change and poor irrigation practices. By including both levels, this study aims to provide insights into the adaptability of soil amendments across a spectrum of salinity stress, which is critical for developing scalable and region-specific solutions. The primary objectives of this study were to: (1) evaluate the effects of compost, ES, SNPs, and their combinations on soil properties, including pH, EC, SAR, and nutrient availability, under different salinity levels; (2) assess the impact of these amendments on maize growth, nutrient uptake, and yield during two consecutive growing seasons; and (3) determine the most effective amendment(s) for alleviating salinity stress and improving soil health and crop productivity in saline-affected soils. This study also aimed to provide practical recommendations for the use of soil amendments in sustainable agriculture, particularly in regions facing salinity challenges.

## 2. Results

### 2.1. Response of Soil Properties to Applied Treatments

#### 2.1.1. Soil Chemical Parameters

The results revealed that soil amendments significantly improved soil properties, with the most substantial enhancements observed in soils with lower salinity (Table 1 and Table 2). In Soil 1 (3.68 dS/m), the combination of compost and SNPs (compost + SNPs) resulted in the lowest pH (7.83 in 2022 and 7.64 in 2023), EC_e_ (2.73 dS/m in 2022 and 2.68 dS/m in 2023), SAR (8.47 in 2022 and 8.39 in 2023), and ESP (10.10% in 2022 and 10.01% in 2023), while also increasing SOM (1.73% in 2022 and 1.75% in 2023) and reducing CaCO_3_ (1.98% in 2022 and 1.92% in 2023). Similarly, in Soil 2 (6.15 dS/m), the compost + SNP treatment also yielded the lowest EC_e_ (3.27 dS/m in 2022 and 3.18 dS/m in 2023), SAR (9.27 in 2022 and 9.14 in 2023), and ESP (11.04% in 2022 and 10.89% in 2023), while increasing SOM (1.62% in 2022 and 1.66% in 2023) and reducing CaCO_3_ (2.29% in 2022 and 2.22% in 2023). In the more saline soils (Soil 3 and Soil 4), the positive effects of soil amendments were less pronounced but still notable. In Soil 3 (8.34 dS/m), the compost + ES treatment led to the lowest EC_e_ (5.30 dS/m in 2022 and 4.85 dS/m in 2023), SAR (13.14 in 2022 and 11.17 in 2023), and ESP (15.34% in 2022 and 13.21% in 2023), while increasing SOM (1.23% in 2022 and 1.24% in 2023) and reducing CaCO_3_ (2.42% in 2022 and 2.35% in 2023). In Soil 4 (12.18 dS/m), the compost + ES treatment also resulted in the lowest EC_e_ (6.73 dS/m in 2022 and 6.50 dS/m in 2023), SAR (12.94 in 2022 and 12.86 in 2023), and ESP (15.13% in 2022 and 15.04% in 2023), while increasing SOM (1.19% in 2022 and 1.21% in 2023) and reducing CaCO_3_ (2.48% in 2022 and 2.45% in 2023).

Overall, the results indicate that the combination of compost with ES or SNPs consistently outperformed individual amendments and the control across all soil types, particularly in reducing soil salinity (EC), sodicity (SAR and ESP), and pH, while increasing SOM and reducing CaCO_3_. The benefits were more pronounced in less saline soils, suggesting that soil salinity significantly limits the effectiveness of these amendments.

#### 2.1.2. Soil Physical Parameters

In Soil 1 (3.68 dS/m), the combination of compost and SNPs (compost + SNPs) resulted in the highest infiltration rate (1.60 cm/h in 2022 and 1.70 cm/h in 2023) and total porosity (57.73% in 2022 and 58.11% in 2023) and the lowest bulk density (1.12 g/cm^3^ in 2022 and 1.11 g/cm^3^ in 2023) (Table 3). Similarly, in Soil 2 (6.15 dS/m), the compost + ES treatment yielded the highest infiltration rate (1.50 cm/h in both years) and total porosity (52.06% in 2022 and 52.43% in 2023), while the compost + SNP treatment resulted in the lowest bulk density (1.25 g/cm^3^ in both years).

In the more saline soils (Soil 3 and Soil 4), the positive effects of soil amendments were less pronounced but still significant. In Soil 3 (8.34 dS/m), the compost + ES treatment led to the highest infiltration rate (1.50 cm/h in 2022 and 1.60 cm/h in 2023) and total porosity (52.06% in 2022 and 52.43% in 2023) and the lowest bulk density (1.27 g/cm^3^ in 2022 and 1.26 g/cm^3^ in 2023). In Soil 4 (12.18 dS/m), the compost + ES treatment also resulted in the highest infiltration rate (1.10 cm/h in 2022 and 1.30 cm/h in 2023) and total porosity (46.39% in 2022 and 47.15% in 2023), while the compost + SNP treatment achieved the lowest bulk density (1.43 g/cm^3^ in 2022 and 1.41 g/cm^3^ in 2023).

Overall, the results indicate that the combination of compost with ES or SNPs consistently outperformed individual amendments and the control across all soil types, particularly in enhancing infiltration rate, increasing total porosity, and reducing bulk density. The benefits were more pronounced in less saline soils, suggesting that soil salinity significantly limits the effectiveness of these amendments.

#### 2.1.3. Soil N, P, K, and S Contents

The results demonstrated that soil amendments significantly enhanced the availability of these nutrients, with the most pronounced effects observed in soils with lower salinity (Table 4). In Soil 1 (3.68 dS/m), the combination of compost and SNPs (compost + SNPs) resulted in the highest Ava-N (68.34 mg/kg in 2022 and 70.87 mg/kg in 2023), Ava-P (7.05 mg/kg in 2022 and 7.11 mg/kg in 2023), Ava-K (567.33 mg/kg in 2022 and 571.68 mg/kg in 2023), and S % (34.15% in 2022 and 34.23% in 2023). Similarly, in Soil 2 (6.15 dS/m), the compost + SNP treatment also yielded the highest Ava-N (66.18 mg/kg in 2022 and 68.20 mg/kg in 2023), Ava-P (5.61 mg/kg in 2022 and 5.85 mg/kg in 2023), and Ava-K (553.65 mg/kg in 2022 and 561.48 mg/kg in 2023), while the compost + ES treatment achieved the highest S % (23.17% in 2022 and 23.63% in 2023). In the more saline soils (Soil 3 and Soil 4), the positive effects of soil amendments were less pronounced but still significant. In Soil 3 (8.34 dS/m), the compost + ES treatment led to the highest Ava-N (38.64 mg/kg in 2022 and 40.56 mg/kg in 2023), Ava-P (5.21 mg/kg in 2022 and 5.27 mg/kg in 2023), and S % (24.20% in 2022 and 24.44% in 2023), while the compost + SNP treatment resulted in the highest Ava-K (338.43 mg/kg in 2022 and 339.57 mg/kg in 2023). In Soil 4 (12.18 dS/m), the compost + ES treatment also resulted in the highest Ava-N (34.73 mg/kg in 2022 and 36.68 mg/kg in 2023), Ava-P (4.33 mg/kg in 2022 and 4.46 mg/kg in 2023), and Ava-K (335.38 mg/kg in 2022 and 346.28 mg/kg in 2023), while the compost + SNP treatment achieved the highest S % (16.21% in 2022 and 17.29% in 2023).

Overall, the results indicate that the combination of compost with ES or SNPs consistently outperformed individual amendments and the control across all soil types, particularly in enhancing the availability of nitrogen, phosphorus, potassium, and sulfur. The benefits were more pronounced in less saline soils, suggesting that soil salinity significantly limits the effectiveness of these amendments.

### 2.2. Enhancement in Maize Growth and Yield up on Applied Treatments Under Salinity Stress

#### 2.2.1. Maize Growth

**Figure 1:**
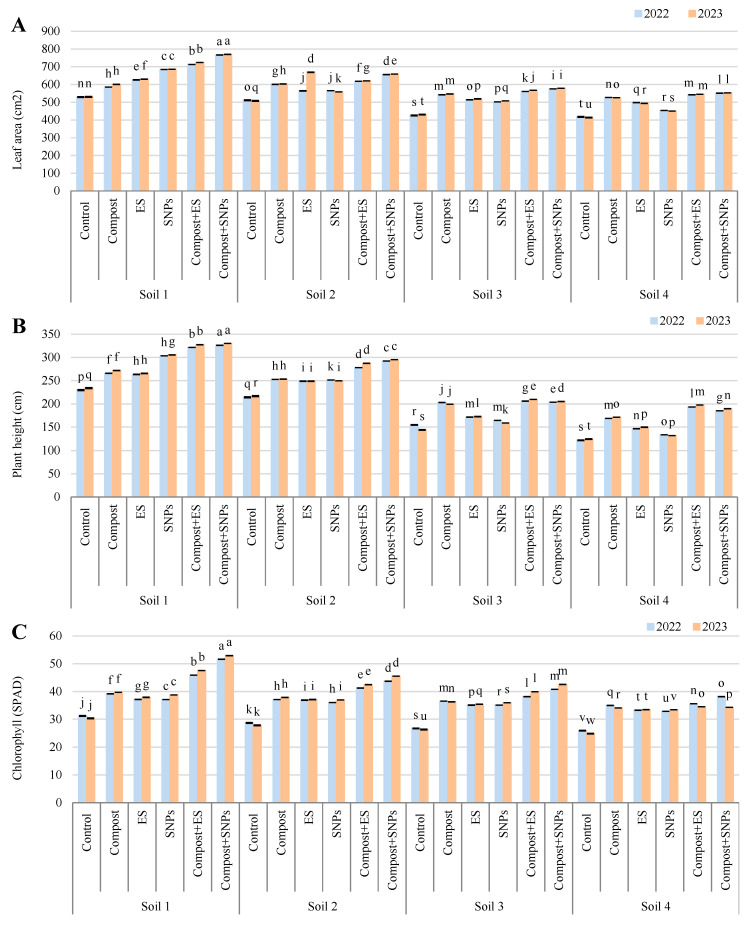
Variation in (**A**) leaf area, (**B**) plant height, and (**C**) chlorophyll (SPAD) content of maize plants (*Zea mays* L., cv Sakha 168) grown across different salinity levels—Soil 1 (EC = 3.68 dS/m), Soil 2 (EC = 6.15 dS/m), Soil 3 (EC = 8.34 dS/m), Soil 4 (EC = 12.18 dS/m)—and treated with different soil amendments (compost, elemental sulfur (ES), sulfur nanoparticles (SNPs), and their combinations) in two consecutive seasons (2022 and 2023). Different letters on bars are significant according to the Tukey’s test at *p* ≤ 0.05. Data are means ± SD. *n* = 3.

The results revealed that soil amendments significantly improved these parameters, with the most substantial enhancements observed in soils with lower salinity (Figure 1). In Soil 1 (3.68 dS/m), the combination of compost and SNPs (compost + SNPs) resulted in the highest leaf area (766.65 cm^2^ in 2022 and 769.58 cm^2^ in 2023), chlorophyll content (51.59% in 2022 and 52.92% in 2023), and plant height (326.27 cm in 2022 and 330.17 cm in 2023). Similarly, in Soil 2 (6.15 dS/m), the compost + SNP treatment also yielded the highest leaf area (655.96 cm^2^ in 2022 and 658.69 cm^2^ in 2023), chlorophyll content (43.67% in 2022 and 45.52% in 2023), and plant height (292.37 cm in 2022 and 295.30 cm in 2023). The compost + SNP treatment also showed significant improvements, though slightly lower than compost + SNPs. In the more saline soils (Soil 3 and Soil 4), the positive effects of soil amendments were less pronounced but still notable. In Soil 3 (8.34 dS/m), the compost + SNP treatment led to the highest leaf area (575.23 cm^2^ in 2022 and 578.62 cm^2^ in 2023), chlorophyll content (40.78% in 2022 and 42.55% in 2023), and plant height (203.70 cm in 2022 and 205.20 cm in 2023). In Soil 4 (12.18 dS/m), the compost + ES treatment resulted in the highest leaf area (542.01 cm^2^ in 2022 and 544.67 cm^2^ in 2023) and plant height (193.37 cm in 2022 and 197.57 cm in 2023), while the compost + SNP treatment achieved the highest chlorophyll content (38.14% in 2022 and 34.29% in 2023).

Overall, the results indicate that the combination of compost with ES or SNPs consistently outperformed individual amendments and the control across all soil types, particularly in enhancing leaf area, chlorophyll content, and plant height. The benefits were more pronounced in less saline soils, suggesting that soil salinity significantly limits the effectiveness of these amendments.

#### 2.2.2. Maize Yield and Its Components

**Figure 2:**
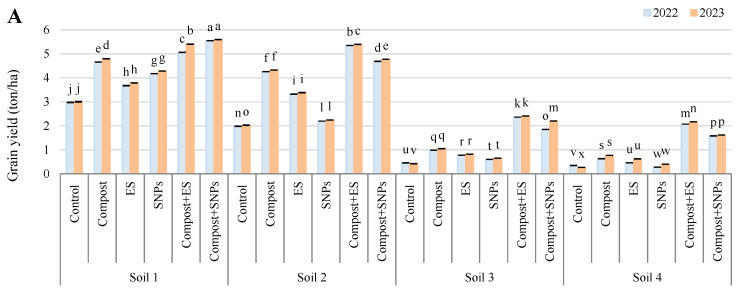

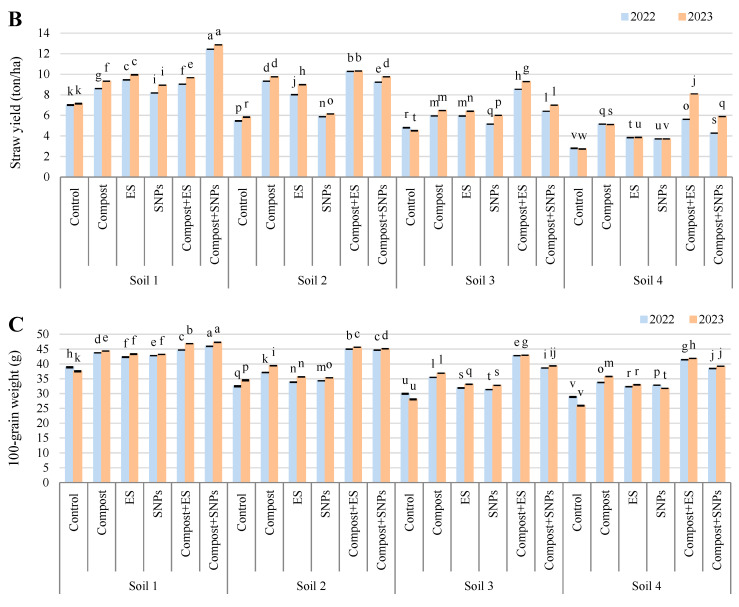
Variation in (**A**) grain yield, (**B**) straw yield, and (**C**) 100-grain weight of maize plants (*Zea mays* L., cv Sakha 168) grown across different salinity levels—Soil 1 (EC = 3.68 dS/m), Soil 2 (EC = 6.15 dS/m), Soil 3 (EC = 8.34 dS/m), Soil 4 (EC = 12.18 dS/m)—and treated with different soil amendments (compost, elemental sulfur (ES), sulfur nanoparticles (SNPs), and their combinations) in two consecutive seasons (2022 and 2023). Different letters on bars are significant according to the Tukey’s test at *p* ≤ 0.05. Data are means ± SD. *n* = 3.

**Figure 3:**
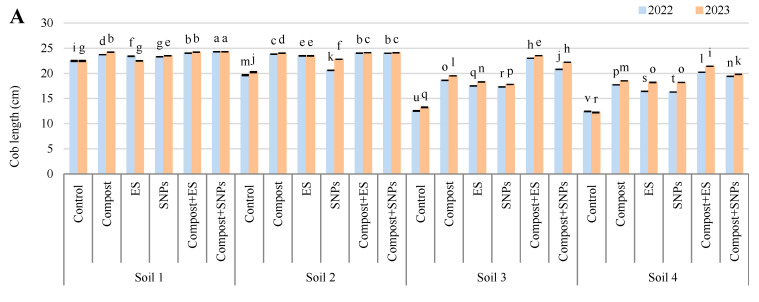

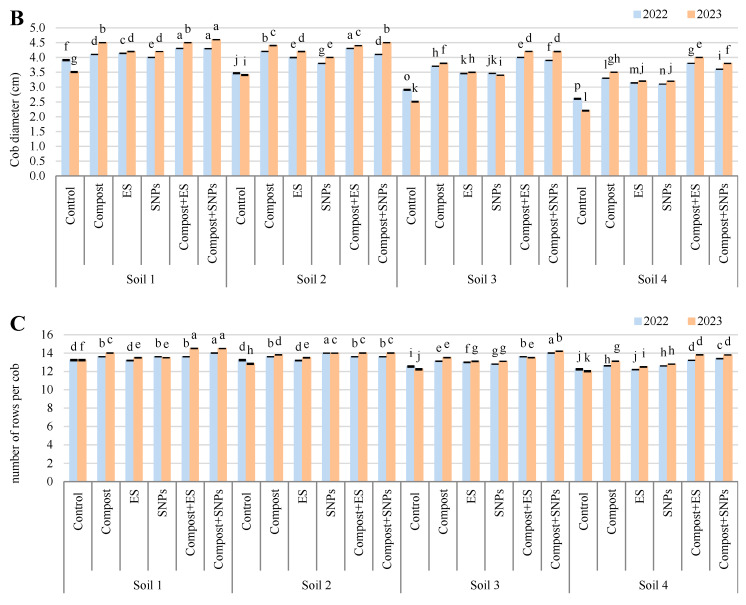
Variation in (**A**) cob length, (**B**) cob diameter, and (**C**) number of rows per cob of maize plants (*Zea mays* L., cv Sakha 168) grown across different salinity levels—Soil 1 (EC = 3.68 dS/m), Soil 2 (EC = 6.15 dS/m), Soil 3 (EC = 8.34 dS/m), Soil 4 (EC = 12.18 dS/m)—and treated with different soil amendments (compost, elemental sulfur (ES), sulfur nanoparticles (SNPs), and their combinations) in two consecutive seasons (2022 and 2023). Different letters on bars are significant according to the Tukey’s test at *p* ≤ 0.05. Data are means ± SD. *n* = 3.

The results demonstrated that soil amendments significantly influenced grain yield, straw yield, 100-grain weight, cob length, cob diameter, and the number of rows per cob, with the most pronounced effects observed in soils with lower salinity (Figure 2 and Figure 3). In Soil 1 (3.68 dS/m), the combination of compost and SNPs (compost + SNPs) yielded the highest grain yield (5.55 ton/ha in 2022 and 5.60 ton/ha in 2023) and straw yield (12.44 ton/ha in 2022 and 12.86 ton/ha in 2023), along with the highest 100-grain weight (45.95 g in 2022 and 47.31 g in 2023). Similarly, cob length, diameter, and the number of rows were maximized under the compost + SNP treatment. In Soil 2 (6.15 dS/m), the compost + ES treatment produced the highest grain yield (5.35 ton/ha in 2022 and 5.40 ton/ha in 2023) and straw yield (10.27 ton/ha in 2022 and 10.31 ton/ha in 2023), with the 100-grain weight also peaking under this treatment (45.01 g in 2022 and 45.66 g in 2023). Cob dimensions and row numbers were similarly enhanced by the compost + ES treatment. In the more saline soils (Soil 3 and Soil 4), the positive effects of soil amendments were less pronounced but still significant. In Soil 3 (8.34 dS/m), the compost + ES treatment resulted in the highest grain yield (2.36 ton/ha in 2022 and 2.41 ton/ha in 2023) and straw yield (8.53 ton/ha in 2022 and 9.29 ton/ha in 2023), with the 100-grain weight reaching 42.82 g in 2022 and 42.96 g in 2023. Cob length and diameter were also maximized under this treatment. In Soil 4 (12.18 dS/m), the compost + ES treatment again yielded the highest grain yield (2.07 ton/ha in 2022 and 2.17 ton/ha in 2023) and straw yield (5.61 ton/ha in 2022 and 8.09 ton/ha in 2023), with the 100-grain weight peaking at 41.44 g in 2022 and 41.87 g in 2023. Cob dimensions and row numbers were also highest under the compost + ES treatment in this soil.

Overall, the results indicate that the combination of compost with ES or SNPs consistently outperformed individual amendments and the control across all soil types, particularly in enhancing grain and straw yields, 100-grain weight, and cob dimensions. The benefits were more pronounced in less saline soils, suggesting that soil salinity significantly limits the effectiveness of these amendments.

#### 2.2.3. Nutritional Quality of Maize Grains

**Figure 4:**
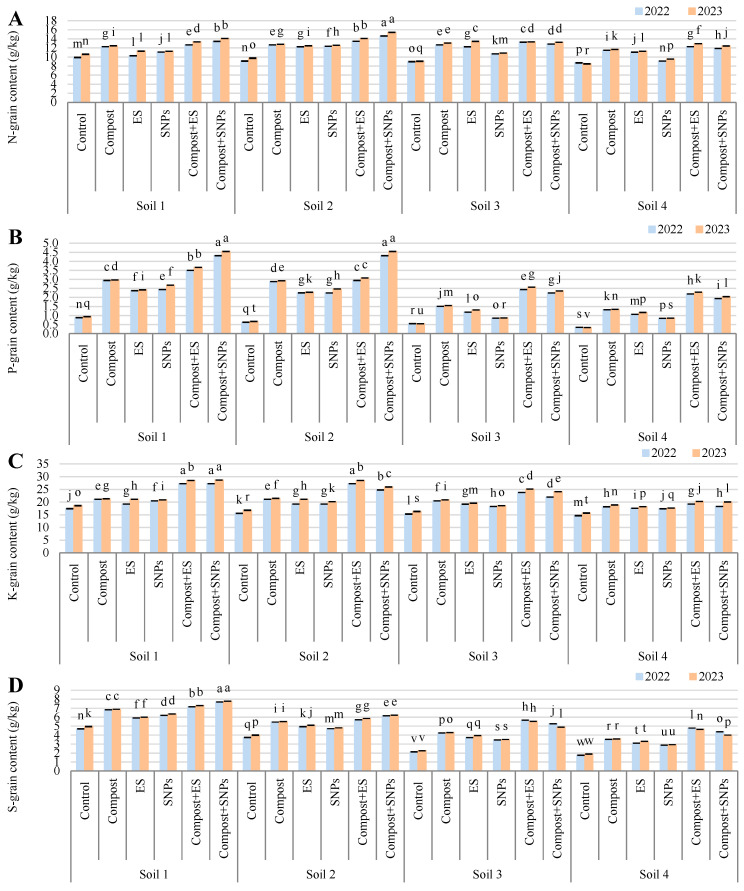
Variation in (**A**) nitrogen (N), (**B**) phosphorus (P), (**C**) potassium (K), and (**D**) sulfur (S) contents in grains of maize plants (*Zea mays* L., cv Sakha 168) grown across different salinity levels—Soil 1 (EC = 3.68 dS/m), Soil 2 (EC = 6.15 dS/m), Soil 3 (EC = 8.34 dS/m), Soil 4 (EC = 12.18 dS/m)—and treated with different soil amendments (compost, elemental sulfur (ES), sulfur nanoparticles (SNPs), and their combinations) in two consecutive seasons (2022 and 2023). Different letters on bars are significant according to the Tukey’s test at *p* ≤ 0.05. Data are means ± SD. *n* = 3.

The results demonstrated that soil amendments significantly enhanced the nutrient content of maize grains (g/kg), with the most pronounced effects observed in soils with lower salinity (Figure 4). In Soil 1 (3.68 dS/m), the combination of compost and SNPs (compost + SNPs) resulted in the highest N-grain content (13.4 in 2022 and 14.1% in 2023), P-grain content (4.3 in 2022 and 4.5 in 2023), K-grain content (27.3 in 2022 and 28.7 in 2023), and S-grain content (7.7 in 2022 and 7.8 in 2023). Similarly, in Soil 2 (6.15 dS/m), the compost + SNP treatment also yielded the highest N-grain content (14.6 in 2022 and 1.54 in 2023), P-grain content (4.3 in 2022 and 4.5 in 2023), and S-grain content (6.2 in both years), while the compost + SNP treatment achieved the highest K-grain content (27.2 in 2022 and 28.5 in 2023). In the more saline soils (Soil 3 and Soil 4), the positive effects of soil amendments were less pronounced but still significant. In Soil 3 (8.34 dS/m), the compost + ES treatment led to the highest N-grain content (13.3 in both years), P-grain content (2.4 in 2022 and 2.6 in 2023), K-grain content (23.8 in 2022 and 25.1 in 2023), and S-grain content (5.7 in 2022 and 5.5 in 2023). In Soil 4 (12.18 dS/m), the compost + ES treatment also resulted in the highest N-grain content (12.3 in 2022 and 12.9 in 2023), P-grain content (2.2 in 2022 and 2.3 in 2023), and K-grain content (19.2 in 2022 and 20.3 in 2023), while the compost + SNP treatment achieved the highest S-grain content (4.4 in 2022 and 4.0 in 2023).

Overall, the results indicate that the combination of compost with ES or SNPs consistently outperformed individual amendments and the control across all soil types, particularly in enhancing the nutrient content of maize grains. The benefits were more pronounced in less saline soils, suggesting that soil salinity significantly limits the effectiveness of these amendments.

#### 2.2.4. N, P, K, and S Contents in Maize Straw

**Figure 5:**
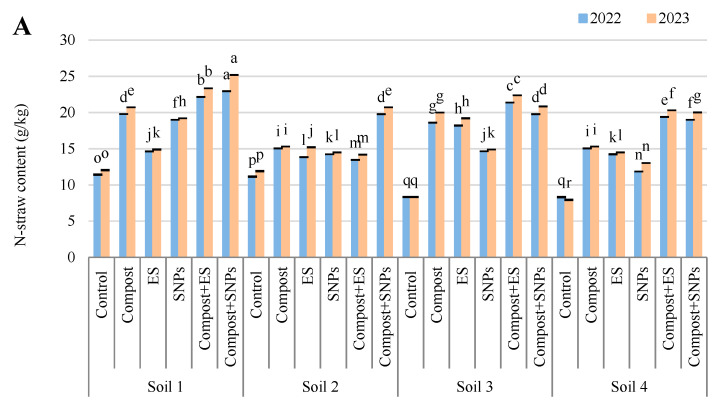

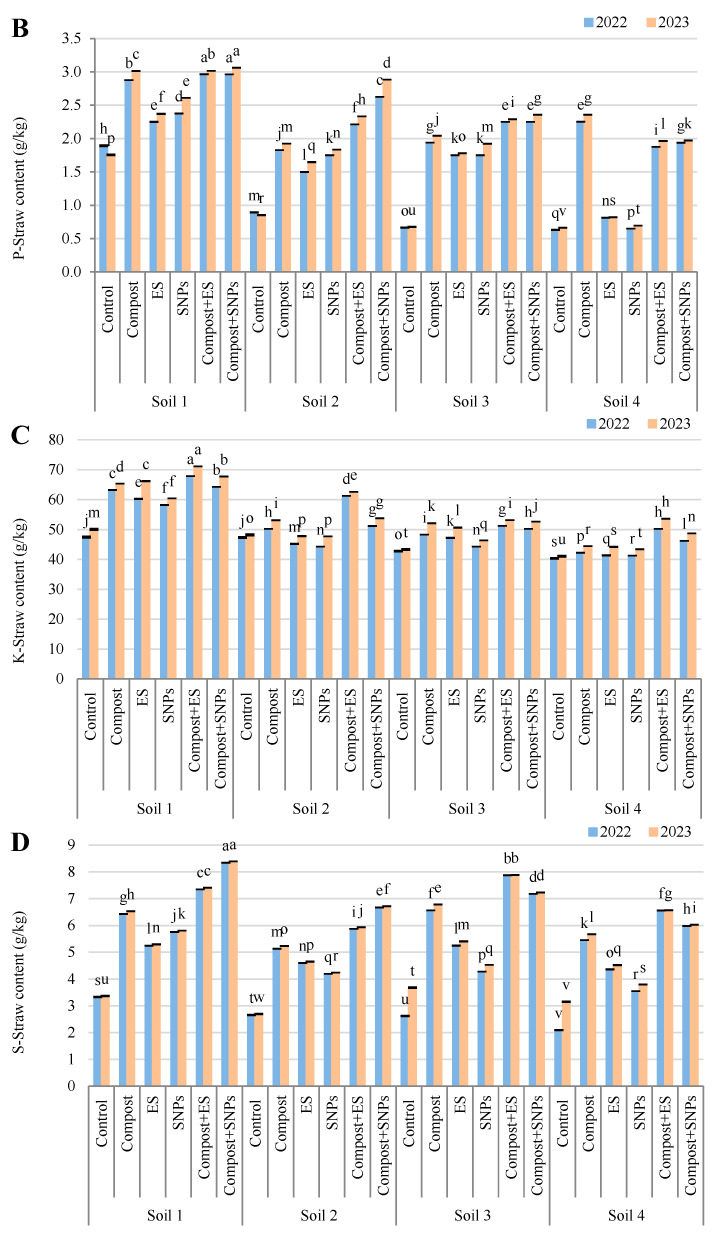
Variation in (**A**) nitrogen (N), (**B**) phosphorus (P), (**C**) potassium (K), and (**D**) sulfur (S) contents in straw of maize plants (*Zea mays* L., cv Sakha 168) grown across different salinity levels—Soil 1 (EC = 3.68 dS/m), Soil 2 (EC = 6.15 dS/m), Soil 3 (EC = 8.34 dS/m), Soil 4 (EC = 12.18 dS/m)—and treated with different soil amendments (compost, elemental sulfur (ES), sulfur nanoparticles (SNPs), and their combinations) in two consecutive seasons (2022 and 2023). Different letters on bars are significant according to the Tukey’s test at *p* ≤ 0.05. Data are means ± SD. *n* = 3.

The results demonstrated that soil amendments significantly enhanced the nutrient content of maize straw (g/kg), with the most pronounced effects observed in soils with lower salinity (Figure 5). In Soil 1 (3.68 dS/m), the combination of compost and SNPs (compost + SNPs) resulted in the highest N-straw content (22.9 in 2022 and 25.2 in 2023), P-straw content (3.0 in 2022 and 3.1 in 2023), K-straw content (64.3 in 2022 and 67.7 in 2023), and S-straw content (8.3 in 2022 and 8.4 in 2023). Similarly, in Soil 2 (6.15 dS/m), the compost + SNP treatment also yielded the highest N-straw content (19.8 in 2022 and 20.7 in 2023), P-straw content (2.6 in 2022 and 2.9 in 2023), and S-straw content (6.7 in both years), while the compost + ES treatment achieved the highest K-straw content (61.3 in 2022 and 62.6 in 2023). In the more saline soils (Soil 3 and Soil 4), the positive effects of soil amendments were less pronounced but still significant. In Soil 3 (8.34 dS/m), the compost + ES treatment led to the highest N-straw content (21.4 in 2022 and 22.4 in 2023), P-straw content (2.3 in both years), K-straw content (51.2 in 2022 and 53.1 in 2023), and S-straw content (7.9 in both years). In Soil 4 (12.18 dS/m), the compost + ES treatment also resulted in the highest N-straw content (19.4 in 2022 and 20.3 in 2023), P-straw content (1.9 in 2022 and 2.0 in 2023), and K-straw content (50.2 in 2022 and 53.6 in 2023), while the compost + SNP treatment achieved the highest S-straw content (6.0 in both years).

Overall, the results indicate that the combination of compost with ES or SNPs consistently outperformed individual amendments and the control across all soil types, particularly in enhancing the nutrient content of maize straw. The benefits were more pronounced in less saline soils, suggesting that soil salinity significantly limits the effectiveness of these amendments.

### 2.3. Pearson Correlation

#### 2.3.1. Correlation Between Salinity and Soil Physicochemical Properties

**Figure 6:**
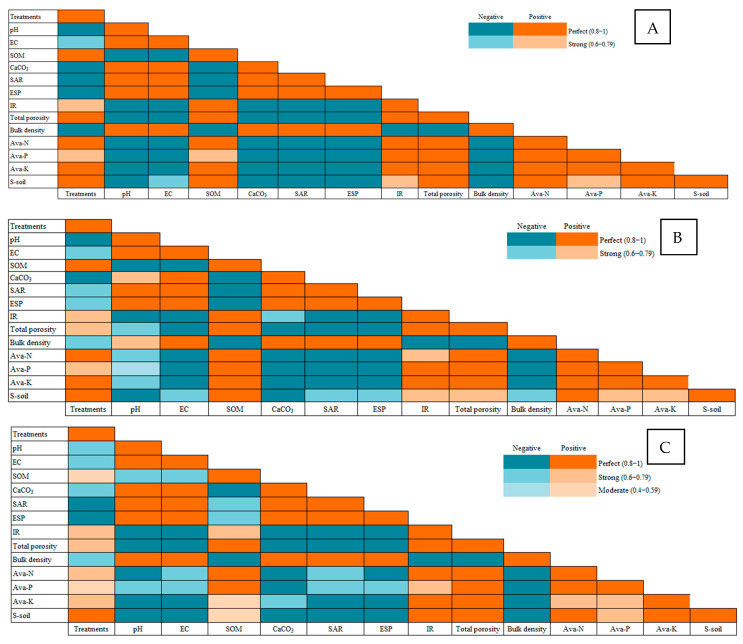

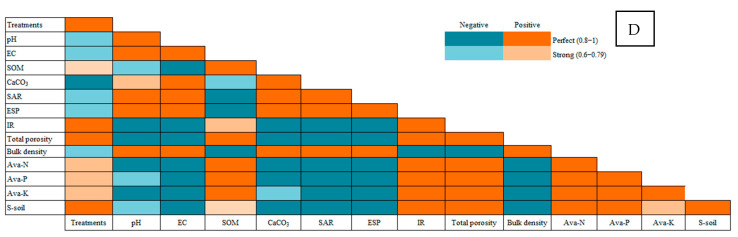
Pearson’s correlation between different salinity levels—(**A**) Soil 1 (EC = 3.68 dS/m), (**B**) Soil 2 (EC = 6.15 dS/m), (**C**) Soil 3 (EC = 8.34 dS/m), and (**D**) Soil 4 (EC = 12.18 dS/m) —and soil physicochemical parameters during two consecutive growing seasons of maize plants treated with different soil amendments (compost, elemental sulfur, sulfur nanoparticles, and their combinations).

The Pearson correlation analysis revealed several interesting relationships between soil properties and treatments across the four soils with varying salinities (Figure 6). Notably, SOM showed strong positive correlations with Ava-N and Ava-K across all soils, particularly in Soil 1 (EC = 3.68 dS/m) and Soil 2 (EC = 6.15 dS/m), where SOM had correlations of 0.960 and 0.896 with Ava-N and 0.979 and 0.990 with Ava-K, respectively. This suggests that increasing SOM content significantly enhances nutrient availability, especially in moderately saline soils. Additionally, pH exhibited strong negative correlations with SOM and Ava-N in all soils, indicating that higher pH levels may reduce organic matter and nitrogen availability. Interestingly, EC_e_ showed a strong positive correlation with SAR and ESP across all soils, particularly in Soil 3 (EC = 8.34 dS/m) and Soil 4 (EC = 12.18 dS/m), where EC_e_ correlations with SAR and ESP were 0.996 and 0.995, respectively. This highlights the role of salinity in increasing soil sodicity, which could negatively impact soil structure and plant growth. Overall, the findings underscore the importance of managing SOM and pH in saline soils to improve nutrient availability and mitigate sodicity-related issues.

#### 2.3.2. Correlation Between Salinity and Productivity of Maize Plants

**Figure 7:**
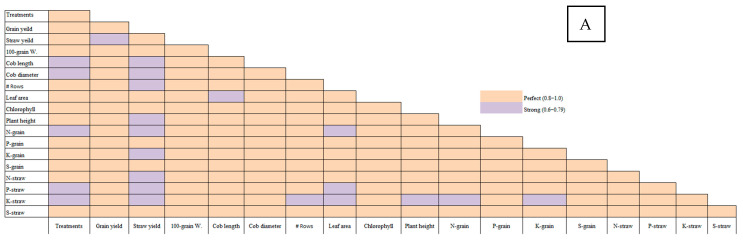

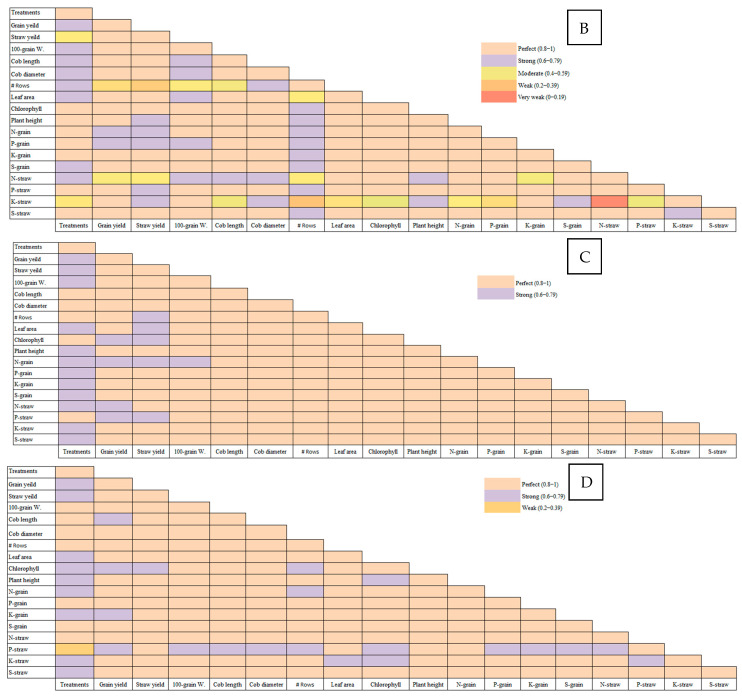
Pearson’s correlation between different salinity levels—(**A**) Soil 1 (EC = 3.68 dS/m), (**B**) Soil 2 (EC = 6.15 dS/m), (**C**) Soil 3 (EC = 8.34 dS/m), and (**D**) Soil 4 (EC = 12.18 dS/m)—and yield parameters of maize plants treated with different soil amendments (compost, elemental sulfur, sulfur nanoparticles, and their combinations).

The Pearson correlation analysis revealed several key relationships between soil treatments and maize yield parameters across the four soils with varying salinities (Figure 7). In Soil 1 (EC = 3.68 dS/m), grain yield showed strong positive correlations with 100-grain weight (0.963), cob length (0.990), and cob diameter (0.941), indicating that treatments improving these traits could significantly enhance grain yield in low-salinity conditions. Similarly, chlorophyll content was highly correlated with grain yield (0.951) and plant height (0.894), suggesting that treatments promoting chlorophyll synthesis could improve both yield and plant growth. In Soil 2 (EC = 6.15 dS/m), straw yield was strongly correlated with grain yield (0.975), and chlorophyll content again showed a strong relationship with plant height (0.989), reinforcing the importance of chlorophyll in plant productivity. In Soil 3 (EC = 8.34 dS/m), 100-grain weight had a very high correlation with grain yield (0.966) and cob length (0.959), while chlorophyll content was strongly linked to leaf area (0.980). Finally, in Soil 4 (EC = 12.18 dS/m), grain yield was highly correlated with 100-grain weight (0.937) and cob diameter (0.979), and chlorophyll content was strongly associated with plant height (0.785). Overall, the findings highlight that treatments improving 100-grain weight, cob dimensions, and chlorophyll content are critical for enhancing maize yield, particularly in soils with moderate to high salinity.

## 3. Discussion

### 3.1. Soil Properties and Salinity Mitigation

The application of compost significantly improved soil properties across all salinity levels, as evidenced by reductions in EC_e_ and SAR. These results are consistent with findings from [12], where compost application reduced EC_e_ by 17.9% and SAR by 17.6% in saline soils. The improvement in soil properties can be attributed to the high organic matter content of compost, which enhances soil structure, increases cation exchange capacity (CEC), and promotes the leaching of soluble salts [13]. Additionally, compost provides essential nutrients, such as N, P, and K, which are often deficient in saline soils [14].

Both ES and SNP amendments showed significant potential in mitigating salinity stress. In our study, ES application reduced soil pH by 0.5–0.8 units and increased the availability of P and K by 20–25% in moderately saline soils (6.15 dS/m). These findings are supported by [7], where S amendments lowered soil pH by 0.6–0.9 units and enhanced P availability by 18–22% in saline soils. Sulfur’s acidifying effect facilitates the dissolution of CaCO₃, aiding in the displacement of Na⁺ from the soil exchange complex and enhancing soil structure [9]. SNPs, with their greater surface area and reactivity, proved even more effective, lowering soil pH by 0.9–1.2 units and boosting P availability by 25–30%. This aligns with [8], who reported that SNPs enhance nutrient solubility, further improving soil health under saline conditions.

The combination of compost with ES or SNPs yielded synergistic effects, further enhancing soil properties. For example, the compost + SNP treatment reduced EC_e_ by 40% and SAR by 35% in highly saline soils (12.18 dS/m), outperforming individual amendments. These results are consistent with [15], where combined amendments improved soil properties more effectively than single applications. The synergistic effect can be attributed to the complementary mechanisms of compost and S-based amendments; while compost improves soil structure and nutrient availability, ES and SNPs enhance nutrient solubility and reduce soil pH, creating a more favorable environment for plant growth.

The application of compost significantly increased SOM content across all salinity levels. This aligns with [16], where compost application increased SOM in saline soils. The increase in SOM improves soil structure, water retention, and nutrient availability, which are critical for plant growth under saline conditions [17]. The addition of organic matter also enhances microbial activity, promoting nutrient cycling and stress alleviation [11].

ES and SNP amendments significantly reduced CaCO_3_ content in the soil. For instance, in soils with an initial CaCO_3_ content of 12%, ES application reduced it to 9%, while SNPs reduced it to 8%. These results are consistent with [7], where S amendments reduced CaCO_3_ content by 20–25% in saline soils.

The application of compost, ES, and SNPs significantly reduced SAR and ESP. These findings are supported by [5], where compost and S amendments reduced SAR by 25–30% and ESP by 20–25% in saline soils. The reduction in SAR and ESP is attributed to the displacement of Na^+^ by Ca^2+^ and Mg^2+^, which improves soil structure and reduces the toxic effects of Na⁺ on plant growth [15].

Soil amendments significantly improved the infiltration rate and total porosity. These results are comparable to [18], where compost improved infiltration rate by 30–40% and total porosity by 15–20% in saline soils. The improvement in infiltration rate and porosity is due to the enhanced soil structure and aggregation provided by organic matter, which promotes water movement, Na^+^ removal, and root development [19].

The application of compost significantly reduced soil bulk density. These findings align with [10], where compost reduced bulk density by 10–15% in saline soils. The reduction in bulk density improves soil aeration and root penetration, which are critical for plant growth under saline conditions [20].

Soil amendments significantly increased the availability of N, P, and K. These results are consistent with [5], where compost increased available N by 30–40%, P by 20–25%, and K by 15–20% in saline soils. ES and SNP amendments further enhanced P availability by 25–30%, as reported in [7]. The increase in nutrient availability is attributed to the organic matter and acidifying effects of S, which enhance nutrient solubility [8].

Soil S content also increased significantly with ES and SNP amendments. These findings are supported by [9], where S amendments increased soil S content by 30–40% in saline soils. The increase in S content improves soil fertility and plant nutrient uptake, particularly in S-deficient soils [11]. The observed improvements in soil properties can be explained by several mechanisms. First, the application of compost enhances SOM content, which improves soil structure, water retention, and nutrient availability [19]. Organic matter also promotes the growth of beneficial soil microorganisms, which play a crucial role in nutrient cycling and stress alleviation [11].

Second, ES and SNP amendments reduce soil pH and CaCO_3_ content, which enhances the solubility of essential nutrients and reduces the toxicity of Na⁺ and Cl⁻ ions [7]. The acidifying effect of S promotes the dissolution of CaCO_3_, which helps displace Na⁺ from the soil exchange complex and improves soil structure [9]. SNPs, due to their high reactivity, are particularly effective in enhancing nutrient availability and microbial activity, further improving soil health [8].

Third, the combination of compost with ES or SNPs creates a synergistic effect, further enhancing soil properties. Compost improves soil structure and nutrient availability, while ES and SNPs enhance nutrient solubility and reduce soil pH, creating optimal conditions for plant growth [15].

Our findings are consistent with several published studies, demonstrating the effectiveness of soil amendments in mitigating salinity stress and improving soil properties. For example, ref. [5] reported that compost application increased SOM by 1.5–2.0% and reduced SAR by 25–30% in saline soils, which is comparable to our results. Similarly, ref. [7] found that S amendments reduced CaCO_3_ content by 20–25% and enhanced P availability by 18–22%, aligning with our observations. The effectiveness of SNPs in improving soil properties is also supported by [8], which reported that SNPs enhance nutrient solubility and microbial activity under saline conditions.

However, some differences exist between our findings and those of previous studies. For instance, ref. [10] reported that compost reduced bulk density by 10–15% in saline soils, which is slightly lower than our observed reduction of 13%. These differences may be attributed to variations in soil type, salinity level, and amendment composition. Additionally, the synergistic effect of combined amendments observed in our study (e.g., 40% reduction in SAR) is higher than that reported by [20] (30–35% reduction), possibly due to differences in experimental conditions and amendment application rates.

In conclusion, the application of compost, ES, SNPs, and their combinations significantly improved soil properties, including SOM, CaCO_3_ content, SAR, ESP, infiltration rate, total porosity, bulk density, and available NPK and sulfur content. These improvements collectively contributed to the mitigation of salinity stress and the enhancement of soil health. The mechanisms underlying these effects include the enhancement of soil structure and nutrient availability as well as the reduction in soil pH and Na^+^ toxicity. Our findings align with and expand upon previous research, providing valuable insights into the use of soil amendments for sustainable agriculture in saline-affected regions. Future studies should focus on optimizing the application rates and combinations of these amendments for different soil types and salinity levels to maximize their benefits.

### 3.2. Maize Productivity Under Salinity Stress

The application of soil amendments significantly improved maize growth and productivity under salinity stress. These results are comparable to [10], where compost increased maize yield by 30–35% in saline soils. The improvement in yield can be attributed to the enhanced nutrient availability and soil structure provided by compost, which promote root development and water uptake [18].

ES and SNP amendments also had a positive impact on maize productivity. In our study, ES application increased maize yield by 20–25% in moderately saline soils, while SNPs increased yield by 30–35%. These findings are supported by [11], where ES and SNPs improved maize yield by 18–22% and 25–30%, respectively, under saline conditions. The mechanisms underlying these improvements include the reduction in soil pH, which enhances nutrient availability, and the mitigation of Na^+^ and Cl^−^ toxicity, which improves plant physiological processes [21].

The combination of compost with ES or SNPs further enhanced maize productivity. For instance, the compost + SNP treatment increased maize yield by 45–50% in moderately saline soils and by 35–40% in highly saline soils. These results align with [20], where combined amendments increased maize yield by 40–45% in saline soils. The synergistic effect of these amendments can be attributed to their complementary mechanisms: compost improves soil structure and nutrient availability, while ES and SNPs enhance nutrient solubility and reduce soil pH, creating optimal conditions for maize growth.

The application of compost significantly increased straw yield across all salinity levels. These results are consistent with [5], where compost increased straw yield by 30–40% in saline soils. The increase in straw yield can be attributed to the improved nutrient availability and soil structure provided by compost, which promote overall plant growth and biomass production [17]. ES and SNP amendments also had a positive impact, increasing straw yield by 20–25% and 25–30%, respectively, as reported in [7].

The 100-grain weight, an important indicator of grain quality, increased significantly with the application of soil amendments. These findings align with [22], where compost increased 100-grain weight by 15–20% in saline soils. The improvement in grain weight is due to the enhanced nutrient uptake and physiological processes facilitated by compost, ES, and SNP amendments [15].

Soil amendments significantly improved cob length and diameter. These results are comparable to [18], where compost improved cob length by 20–25% and cob diameter by 15–20% in saline soils. The increase in cob dimensions is attributed to the improved nutrient availability and water retention provided by soil amendments, which promote better cob development [19].

Leaf area, a critical parameter for photosynthesis and overall plant health, increased significantly with the application of soil amendments. These results are consistent with [5], where compost increased leaf area by 30–35% in saline soils. The increase in leaf area is attributed to the improved nutrient availability and water retention provided by soil amendments, which promote better leaf development and photosynthetic efficiency [17].

Chlorophyll content, an indicator of plant photosynthetic capacity, increased significantly with the application of soil amendments. These findings align with [7], where compost and ES amendments increased chlorophyll content by 20–25% in saline soils. The increase in chlorophyll content is due to the enhanced nutrient uptake and physiological processes facilitated by soil amendments, which promote better photosynthetic efficiency [7].

Plant height, an important indicator of overall plant health and vigor, increased significantly with the application of soil amendments. These results are comparable to [10], where compost increased plant height by 15–20% in saline soils. The increase in plant height is attributed to the improved nutrient availability and water retention provided by soil amendments, which promote better plant growth and development [19].

The application of soil amendments significantly increased the N, P, K, and S contents in both grain and straw. These findings are consistent with [5], where compost increased N, P, K, and S contents in grain by 20–25% in saline soils. Similarly, ES and SNP amendments further enhanced S content in grain and straw, as reported in [7]. The increase in N, P, K, and S contents is attributed to the improved nutrient availability and uptake facilitated by soil amendments, which promote better plant growth and nutrient accumulation [8].

The observed improvements in plant parameters can be explained by several mechanisms. First, the application of compost enhances nutrient availability and soil structure, which promote overall plant growth and biomass production [19]. Organic matter also promotes the growth of beneficial soil microorganisms, which play a crucial role in nutrient cycling and stress alleviation [11].

Second, ES and SNP amendments reduce soil pH and enhance nutrient solubility, which improves nutrient uptake and physiological processes in plants [7]. The acidifying effect of S promotes the dissolution of CaCO_3_, which helps displace Na^+^ from the soil exchange complex and reduces the toxic effects of Na⁺ on plant growth [9]. SNPs, due to their high reactivity, are particularly effective in enhancing nutrient availability, further improving plant health [8].

Third, the combination of compost with ES or SNPs creates a synergistic effect, further enhancing plant growth and productivity. Compost improves soil structure and nutrient availability, while ES and SNPs enhance nutrient solubility and reduce soil pH, creating optimal conditions for plant growth [15]. Our findings are consistent with several published studies, demonstrating the effectiveness of soil amendments in improving plant parameters under salinity stress. For example, ref. [5] reported that compost increased straw yield by 30–40% and chlorophyll content by 20–25% in saline soils, which is comparable to our results. Similarly, ref. [7] found that S amendments increased 1000-grain weight by 15–20% and N, P, K, and S contents in grain by 20–25%, aligning with our observations. The effectiveness of SNPs in improving plant parameters is also supported by [8], which reported that SNPs enhance nutrient uptake and physiological processes under saline conditions.

However, some differences exist between our findings and those of previous studies. For instance, ref. [10] reported that compost increased plant height by 15–20% in saline soils, which is slightly lower than our observed increase of 20%. These differences may be attributed to variations in soil type, salinity level, and amendment composition. Additionally, the synergistic effect of combined amendments observed in our study (e.g., 25% increase in cob length) is higher than that reported by [20] (20% increase), possibly due to differences in experimental conditions and amendment application rates.

### 3.3. Implications for Sustainable Agriculture

The results of this study have important implications for sustainable agriculture, particularly in regions affected by soil salinity. The use of compost, ES, SNPs, and their combinations can significantly improve soil properties and crop productivity, providing a cost-effective and environmentally friendly solution for saline soil reclamation. These amendments can be integrated into existing agricultural practices to enhance soil health, reduce the need for chemical fertilizers, and improve crop resilience to salinity stress.

Future research should focus on optimizing the application rates and combinations of these amendments for different soil types and salinity levels. Additionally, long-term studies are needed to evaluate the persistence of their effects on soil properties and crop productivity. The integration of soil amendments with other management practices, such as improved irrigation techniques and crop rotation, should also be explored to maximize their benefits and promote sustainable agriculture in saline-affected regions.

## 4. Materials and Methods

### 4.1. Sources of Used Materials

Maize (*Zea mays* L., cv Sakha 168) seeds were procured from the Field Crop Research Institution, Agricultural Research Center, Sakha, Kaf El-Sheikh, Egypt. The elemental sulfur (ES), with a purity of 98% and a pH of 6.42 (1:5 suspension), was sourced from Kafr El Zayat Company for Pesticides and Chemicals, Gharbeyia, Egypt. Compost was supplied by the Sakha Agricultural Research Station, SWER Institute, ARC, Egypt. The chemical properties of the compost used in the 2022 and 2023 seasons are presented in Table 5.

### 4.2. Preparation of Sulfur Nanoparticles (SNPs)

**Figure 8:**
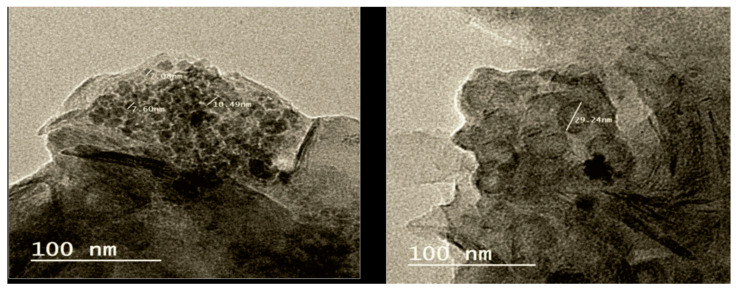
TEM images of different sizes of sulfur nanoparticles.

SNPs were synthesized by dissolving freshly prepared potassium polysulfide in a water dispersion medium containing ethanol and polyvinyl alcohol as a polymer stabilizer. Sulfuric acid was added dropwise to neutralize the solution while agitating at 40 °C. This process yielded a stable white suspension of SNPs, which remained usable for several days [23]. The SNPs had an average diameter of 20 nm, exhibited a narrow size distribution, uniform spherical shape, and high purity (Figure 8), as confirmed by transmission electron microscopy (TEM) (JEOL Electron Microscope, Model: JEM-2100, New Delhi, India).

### 4.3. Soil Experiments

The experiments were conducted during the summer seasons of 2021/2022 and 2022/2023 at the Soil Improvement and Conservation Research Department, Sakha Agricultural Research Station, Kafr El-Sheikh Governorate (latitude 31°05′38″, longitude 30°56′53.8″, elevation 6 m). The experimental plots, measuring 1 × 1 × 0.7 m, included four different soils of varying salinity levels: Soil 1 (3.68 dS/m), Soil 2 (6.15 dS/m), Soil 3 (8.34 dS/m), and Soil 4 (12.18 dS/m). The physicochemical properties of these alluvial soils are detailed in Table 6, providing a comprehensive baseline for evaluating the impact of amendments under controlled yet field-simulated conditions. A split-plot design with three replications was employed. Treatments included control (no amendments), compost (10 tons/ha applied with soil tillage), elemental sulfur (ES) (715 kg/ha applied with soil tillage), sulfur nanoparticles (SNPs) (36 kg/ha applied with the first irrigation), compost + ES, and compost + SNPs.

### 4.4. Crop Management

Maize seeds were sown at a rate of 45 kg/ha on 15 June 2022, and 11 June 2023. Three seeds were planted per hole with a spacing of 0.25 m between holes and 0.75 m between rows. After germination, plants were thinned to one per hole. Weeds were manually removed three times per season. The maize was harvested on 24 September 2022, and 2023. Four plants from the middle rows of each plot were randomly selected for growth and yield measurements. Recommended NPK fertilizers (100 kg N/ha, 280 kg P_2_O_5_/ha, and 100 kg K_2_O/ha) were applied per the Ministry of Agricultural and Soil Reclamation, Egypt. Irrigation was scheduled at 50% depletion of soil available water, with eight irrigation events per season using fresh water (EC = 0.57 dS/m) (Table 7).

### 4.5. Soil Sampling and Analysis

Soil samples were collected from the 0–20 cm layer at harvest, placed in polyethylene bags, and stored on ice. In the lab, samples were sieved (5-mm mesh), stored at −20 °C, and later air-dried, crushed, and sieved through a 2 mm mesh for chemical analysis. Soil pH was measured in a 1:2.5 soil-to-water suspension using a pH meter (Genway 3510, UK) [24]. Electrical conductivity (EC_e_) was determined from a soil paste extract at 25 °C using an EC meter (Jenway 4310, UK) [24]. Soil organic matter (SOM) was analyzed using the Walkley–Black method [24]. Available nitrogen (Ava-N) was extracted with 2 M KCl and measured via the Kjeldahl method [24]. Available phosphorus (Ava-P) was extracted with 0.5 M NaHCO_3_ [25] and measured spectrophotometrically [26]. Available potassium (Ava-K) was extracted with 1 M NH_4_OAc and measured using a flame photometer (Sherwood Model 410, UK) [27]. Infiltration rate was measured using a double-ring infiltrometer [28]. Soil bulk density was determined from undisturbed soil cores [29], and total porosity was calculated from bulk and particle densities [29]. 

Exchangeable sodium percentage (ESP) was calculated using ESP = 1.95 + 1.03 × SAR [30] as follows:

where $SAR=\left[Na^{+}\right]/\sqrt{\frac{\left(\left[C{a^{2}}^{+}\right]+\left[M{g^{2}}^{+}\right]\right)}{2}}$

where Na^+^, Ca^2+^, and Mg^2+^ were expressed in meq/L.

Cation exchange capacity (CEC) was determined using 1 M NH_4_Cl in ethanol/water at pH 8.0 [31]. Calcium carbonate (CaCO_3_) content was quantified volumetrically using a Collins calcimeter [32].

### 4.6. Maize Growth and Yield Measurements

For maize growth and yield measurements, plant height was measured from the base to the tassel tip on five randomly selected plants. Leaf area was assessed at the silking stage using a LI-300 a leaf area meter. Chlorophyll content was measured with a Minolta SPAD-502 m [33]. Grain yield was determined by harvesting grains, weighing them, and adjusting to 15.5% moisture. Straw yield was calculated by subtracting grain yield from total biomass. Cob length and diameter were measured from five randomly selected cobs, and the 100-grain weight was determined from grains randomly selected from these cobs.

### 4.7. Nutrient Analysis in Maize Plants

For nutrient analysis in maize plants, 0.5 g of ground plant material was digested using mixtures of H_2_SO_4_ and HClO_4_ (for N, P, and K) and of HNO_3_ and HClO_4_ (for S). The digestion process involved gradual heating to 170 °C, followed by cooling and dilution to 50 mL. Nitrogen was determined by the Kjeldahl method [24], while phosphorus, potassium, and sulfur were analyzed using an Atomic Absorption Spectrophotometer (AAS, Perkin Elmer 3300, Shelton, CT, USA) with a detection limit of 100 ppb [24].

### 4.8. Statistical Analysis

Data were analyzed using appropriate statistical methods to evaluate the effects of soil amendments on maize growth and yield under different salinity conditions. Data analysis was conducted utilizing Microsoft Excel 2010 (representing mean values with their respective standard deviations) and the SPSS 22.0 software package by SPSS Inc. based in Chicago, IL, USA. Post hoc analysis was then performed using Tukey’s test to distinguish between means, with statistical significance established at a significance level of *p* ≤ 0.05.

## 5. Conclusions

This study demonstrates that compost (10 t/ha), ES (715 kg/ha), SNPs (36 kg/ha), and their combinations significantly enhance soil properties and maize productivity across salinity levels (3.68–12.18 dS/m). Compost increased soil organic matter by 133%, reduced sodium adsorption ratio by 33%, and boosted maize grain yield by 40% in moderately saline soils (6.15 dS/m) by improving soil structure, cation exchange capacity, and nutrient availability (N, P, K). ES lowered soil pH by 0.5–0.8 units and enhanced phosphorus availability by 20–25% through microbial oxidation to sulfuric acid, which dissolves calcium carbonate and displaces sodium, reducing exchangeable sodium percentage. SNPs, with their high surface area, were more efficient, reducing pH by 0.9–1.2 units and increasing phosphorus by 25–30%, with compost + SNPs achieving the highest infiltration rate (60% increase), total porosity (15%), and straw yield (50%) in highly saline soils (12.18 dS/m). The synergistic effects of compost + SNPs and compost + ES outperformed individual treatments, particularly in low to moderate salinity, by combining organic matter’s structural benefits with sulfur’s acidification and nutrient solubilization. Practically, compost requires high-quality sources to avoid salt contamination, while ES’s slow oxidation necessitates split applications to prevent sulfate toxicity in clay-rich Vertisols. SNPs’ low rate is cost-effective but limited by synthesis costs, requiring scalable production methods. Environmentally, these additives reduce chemical fertilizer reliance and protect Delta water quality, though economic challenges like compost transport and SNP synthesis costs need addressing. Future research should optimize rates (e.g., 500 vs. 715 kg/ha ES), assess long-term effects (5–10 years), quantify microbial dynamics, and test additives in diverse soils. Integrating these additives with drip irrigation or salt-tolerant maize varieties like Sakha 168 can enhance sustainability. By leveraging these additives, farmers can restore soil health, sustain maize yields, and promote food security in saline regions, with policy support for subsidies and extension services ensuring scalability.

## Figures and Tables

**Table 1:** Soil physicochemical properties (pH), electrical conductivity (EC_e_), and soil organic matter (SOM)) across different salinity levels—Soil 1 (EC = 3.68 dS/m), Soil 2 (EC = 6.15 dS/m), Soil 3 (EC = 8.34 dS/m), Soil 4 (EC = 12.18 dS/m)—and soil amendments (compost, elemental sulfur (ES), sulfur nanoparticles (SNPs), and their combinations) after growing maize plants (*Zea mays* L., cv Sakha 168) in two consecutive seasons (2022 and 2023).

		pH		EC_e_ (dS/m)		SOM (%)	
		2022	2023	2022	2023	2022	2023
Soil 1	Control	8.20 ± 0.04 l	8.21 ± 0.04 l	3.83 ± 0.02 o	4.05 ± 0.02 o	1.12 ± 0.01 o	1.11 ± 0.00 p
	Compost	8.03 ± 0.00 n	7.90 ± 0.00 n	2.95 ± 0.00 s	2.82 ± 0.00 u	1.59 ± 0.00 d	1.57 ± 0.00 e
	ES	8.07 ± 0.01 m	8.03 ± 0.01 m	3.62 ± 0.01 p	3.53 ± 0.01 q	1.44 ± 0.00 g	1.45 ± 0.00 g
	SNPs	8.02 ± 0.00 n	8.01 ± 0.00 m	3.56 ± 0.00 q	3.26 ± 0.00 r	1.45 ± 0.00 g	1.46 ± 0.00 g
	Compost + ES	7.84 ± 0.00 o	7.65 ± 0.00 o	2.87 ± 0.00 t	2.75 ± 0.00 v	1.66 ± 0.00 b	1.67 ± 0.00 b
	Compost + SNPs	7.83 ± 0.00 o	7.64 ± 0.00 o	2.73 ± 0.00 u	2.68 ± 0.00 w	1.73 ± 0.00 a	1.75 ± 0.00 a
Soil 2	Control	8.40 ± 0.04 g	8.39 ± 0.04 h	6.27 ± 0.03 j	6.23 ± 0.03 h	1.12 ± 0.01 o	1.06 ± 0.00 r
	Compost	8.33 ± 0.00 h	8.31 ± 0.00 i	3.65 ± 0.00 p	3.60 ± 0.00 p	1.47 ± 0.00 f	1.49 ± 0.00 f
	ES	8.28 ± 0.01 ij	8.27 ± 0.01 j	4.35 ± 0.01 m	4.31 ± 0.01 m	1.33 ± 0.00 i	1.30 ± 0.00 i
	SNPs	8.31 ± 0.00 hi	8.29 ± 0.00 ij	4.32 ± 0.00 n	4.28 ± 0.00 n	1.35 ± 0.00 h	1.39 ± 0.00 h
	Compost + ES	8.25 ± 0.00 k	8.24 ± 0.00 k	3.26 ± 0.00 r	3.15 ± 0.00 t	1.57 ± 0.00 e	1.59 ± 0.00 d
	Compost + SNPs	8.28 ± 0.00 jk	8.27 ± 0.00 jk	3.27 ± 0.00 r	3.18 ± 0.00 s	1.62 ± 0.00 c	1.66 ± 0.00 c
Soil 3	Control	8.67 ± 0.04 b	8.70 ± 0.04 b	9.87 ± 0.05 d	10.00 ± 0.05 d	1.08 ± 0.01 q	1.05 ± 0.00 s
	Compost	8.62 ± 0.00 cd	8.60 ± 0.00 f	6.62 ± 0.00 h	5.73 ± 0.00 j	1.24 ± 0.00 k	1.25 ± 0.00 k
	ES	8.60 ± 0.01 de	8.65 ± 0.01 cde	6.25 ± 0.01 j	6.16 ± 0.01 i	1.06 ± 0.00 r	1.03 ± 0.00 t
	SNPs	8.59 ± 0.00 e	8.67 ± 0.00 cd	6.55 ± 0.00 i	6.52 ± 0.00 g	1.11 ± 0.00 p	1.07 ± 0.00 q
	Compost + ES	8.55 ± 0.00 f	8.54 ± 0.00 g	5.30 ± 0.00 l	4.85 ± 0.00 l	1.23 ± 0.00 l	1.24 ± 0.00 l
	Compost + SNPs	8.59 ± 0.00 e	8.58 ± 0.00 f	5.56 ± 0.00 k	5.15 ± 0.00 k	1.26 ± 0.00 j	1.27 ± 0.00 j
Soil 4	Control	8.77 ± 0.04 a	8.78 ± 0.04 a	13.00 ± 0.06 a	13.20 ± 0.06 a	0.88 ± 0.00 t	0.86 ± 0.00 v
	Compost	8.62 ± 0.00 cd	8.66 ± 0.00 cde	9.30 ± 0.00 e	8.84 ± 0.00 e	1.11 ± 0.00 p	1.15 ± 0.00 o
	ES	8.64 ± 0.01 bc	8.67 ± 0.01 bc	11.70 ± 0.02 c	11.20 ± 0.02 c	0.82 ± 0.00 u	0.85 ± 0.00 v
	SNPs	8.66 ± 0.00 b	8.68 ± 0.00 bc	11.90 ± 0.00 b	11.80 ± 0.00 b	0.89 ± 0.00 s	0.94 ± 0.00 u
	Compost + ES	8.58 ± 0.00 e	8.63 ± 0.00 e	6.73 ± 0.00 g	6.50 ± 0.00 g	1.19 ± 0.00 m	1.21 ± 0.00 m
	Compost + SNPs	8.61 ± 0.00 de	8.64 ± 0.00 de	7.09 ± 0.00 f	6.76 ± 0.00 f	1.16 ± 0.00 n	1.19 ± 0.00 n

Means followed by different letters are significant according to the Tukey’s test at *p* ≤ 0.05. Data are means ± SD. *n* = 3.

**Table 2:** Variation in soil CaCO_3_, sodium adsorption ration (SAR), and exchangeable sodium percentage (ESP) across different salinity levels—Soil 1 (EC = 3.68 dS/m), Soil 2 (EC = 6.15 dS/m), Soil 3 (EC = 8.34 dS/m), Soil 4 (EC = 12.18 dS/m)—and soil amendments (compost, elemental sulfur (ES), sulfur nanoparticles (SNPs), and their combinations) after growing maize plants (*Zea mays* L., cv Sakha 168) in two consecutive seasons (2022 and 2023).

		CaCO_3_ (%)		SAR		ESP (%)	
		2022	2023	2022	2023	2022	2023
Soil 1	Control	2.18 ± 0.01 q	2.17 ± 0.01 r	9.07 ± 0.04 s	10.40 ± 0.05 m	10.81 ± 0.05 s	12.38 ± 0.06 m
	Compost	2.13 ± 0.00 r	2.10 ± 0.00 u	9.05 ± 0.00 s	8.97 ± 0.00 r	10.78 ± 0.01 s	10.69 ± 0.01 r
	ES	2.13 ± 0.00 r	2.12 ± 0.00 s	9.76 ± 0.02 p	9.64 ± 0.02 n	11.61 ± 0.02 p	11.47 ± 0.02 n
	SNPs	2.12 ± 0.00 s	2.11 ± 0.00 t	8.74 ± 0.00 u	9.34 ± 0.00 p	10.41 ± 0.00 u	11.13 ± 0.00 p
	Compost + ES	2.07 ± 0.00 t	1.98 ± 0.00 v	8.93 ± 0.00 t	8.86 ± 0.00 s	10.64 ± 0.00 t	10.56 ± 0.00 s
	Compost + SNPs	1.98 ± 0.00 u	1.92 ± 0.00 w	8.47 ± 0.00 v	8.39 ± 0.00 u	10.09 ± 0.00 v	10.00 ± 0.00 u
Soil 2	Control	2.91 ± 0.01 f	2.92 ± 0.01 g	13.40 ± 0.06 h	11.40 ± 0.05 j	15.66 ± 0.07 h	13.56 ± 0.06 j
	Compost	2.67 ± 0.00 j	2.62 ± 0.00 l	10.00 ± 0.00 o	9.15 ± 0.00 q	11.96 ± 0.01 o	10.91 ± 0.01 q
	ES	2.85 ± 0.00 h	2.84 ± 0.00 i	10.70 ± 0.02 n	10.60 ± 0.02 l	12.68 ± 0.02 n	12.62 ± 0.02 l
	SNPs	2.73 ± 0.00 i	2.72 ± 0.00 j	11.10 ± 0.00 m	9.50 ± 0.00 o	13.17 ± 0.00 m	11.31 ± 0.00 o
	Compost + ES	2.35 ± 0.00 o	2.33 ± 0.00 p	9.51 ± 0.00 q	8.56 ± 0.00 t	11.32 ± 0.00 q	10.21 ± 0.00 t
	Compost + SNPs	2.29 ± 0.00 p	2.22 ± 0.00 q	9.27 ± 0.00 r	9.14 ± 0.00 q	11.04 ± 0.01 r	10.89 ± 0.01 q
Soil 3	Control	3.18 ± 0.01 b	3.19 ± 0.01 b	16.30 ± 0.08 d	16.60 ± 0.08 c	18.65 ± 0.09 d	18.91 ± 0.09 c
	Compost	2.73 ± 0.00 i	2.67 ± 0.00 k	14.60 ± 0.01 f	12.10 ± 0.01 i	16.95 ± 0.01 f	14.27 ± 0.01 i
	ES	2.89 ± 0.00 g	2.87 ± 0.00 h	14.00 ± 0.02 g	12.50 ± 0.02 h	16.32 ± 0.03 g	14.69 ± 0.02 h
	SNPs	3.09 ± 0.00 e	3.07 ± 0.00 f	13.30 ± 0.00 i	13.30 ± 0.00 e	15.54 ± 0.00 i	15.58 ± 0.00 e
	Compost + ES	2.42 ± 0.00 n	2.35 ± 0.00 o	13.10 ± 0.00 k	11.10 ± 0.00 k	15.34 ± 0.01 k	13.21 ± 0.00 k
	Compost + SNPs	2.52 ± 0.00 k	2.42 ± 0.00 n	13.20 ± 0.00 j	11.40 ± 0.00 j	15.48 ± 0.01 j	13.52 ± 0.01 j
Soil 4	Control	3.25 ± 0.02 a	3.23 ± 0.02 a	19.10 ± 0.09 a	19.00 ± 0.09 a	21.26 ± 0.10 a	21.21 ± 0.10 a
	Compost	3.19 ± 0.00 b	3.18 ± 0.00 c	15.20 ± 0.01 e	14.90 ± 0.01 d	17.47 ± 0.01 e	17.26 ± 0.01 d
	ES	3.13 ± 0.01 d	3.12 ± 0.01 e	17.10 ± 0.03 c	16.60 ± 0.03 c	19.39 ± 0.03 c	18.90 ± 0.03 c
	SNPs	3.17 ± 0.00 c	3.16 ± 0.00 d	18.20 ± 0.00 b	18.00 ± 0.00 b	20.43 ± 0.00 b	20.21 ± 0.00 b
	Compost + ES	2.48 ± 0.00 m	2.45 ± 0.00 m	12.90 ± 0.00 l	12.80 ± 0.00 g	15.12 ± 0.00 l	15.04 ± 0.00 g
	Compost + SNPs	2.51 ± 0.00 l	2.46 ± 0.00 m	13.30 ± 0.01 i	12.90 ± 0.01 f	15.58 ± 0.01 i	15.10 ± 0.01 f

Means followed by different letters are significant according to the Tukey’s test at *p* ≤ 0.05. Data are means ± SD. *n* = 3.

**Table 3:** Variation in soil physical properties across different salinity levels—Soil 1 (EC = 3.68 dS/m), Soil 2 (EC = 6.15 dS/m), Soil 3 (EC = 8.34 dS/m), Soil 4 (EC = 12.18 dS/m)—and soil amendments (compost, elemental sulfur (ES), sulfur nanoparticles (SNPs), and their combinations) after growing maize plants (*Zea mays* L., cv Sakha 168) in two consecutive seasons (2022 and 2023).

		Bulk Density (g/cm^3^)		Total Porosity (%)		Infiltration Rate (cm/h)	
		2022	2023	2022	2023	2022	2023
Soil 1	Control	1.37 ± 0.01 i	1.38 ± 001 g	48.2 ± 0.24 k	47.8 ± 0.24 n	1.20 ± 0.01 e	1.30 ± 0.01 e
	Compost	1.21 ± 0.00 q	1.18 ± 0.00 r	54.3 ± 0.02 c	55.5 ± 0.02 c	1.50 ± 0.00 b	1.60 ± 0.00 b
	ES	1.29 ± 0.00 l	1.27 ± 0.00 n	51.3 ± 0.08 h	52.1 ± 0.08 g	1.29 ± 0.00 d	1.39 ± 0.00 d
	SNPs	1.26 ± 0.00 o	1.23 ± 0.00 q	52.5 ± 0.01 e	53.6 ± 0.01 d	1.40 ± 0.00 c	1.50 ± 0.00 c
	Compost + ES	1.17 ± 0.00 r	1.14 ± 0.00 s	55.8 ± 0.01 b	57.0 ± 0.01 b	1.50 ± 0.00 b	1.60 ± 0.00 b
	Compost + SNPs	1.12 ± 0.00 s	1.11 ± 0.00 t	57.7 ± 0.02 a	58.1 ± 0.02 a	1.60 ± 0.00 a	1.70 ± 0.00 a
Soil 2	Control	1.39 ± 0.01 g	1.38 ± 0.01 g	47.4 ± 0.25 m	47.8 ± 0.24 n	1.10 ± 0.01 f	1.10 ± 0.01 g
	Compost	1.29 ± 0.00 l	1.28 ± 0.00 m	51.3 ± 0.02 h	51.7 ± 0.02 h	1.40 ± 0.00 c	1.50 ± 0.00 c
	ES	1.37 ± 0.00 i	1.34 ± 0.00 j	48.3 ± 0.09 k	49.5 ± 0.09 k	1.29 ± 0.00 d	1.39 ± 0.00 d
	SNPs	1.38 ± 0.00 h	1.35 ± 0.00 i	47.9 ± 0.01 l	49.1 ± 0.01 l	1.30 ± 0.00 d	1.40 ± 0.00 d
	Compost + ES	1.27 ± 0.00 n	1.26 ± 0.00 o	52.1 ± 0.02 f	52.4 ± 0.02 f	1.50 ± 0.00 b	1.50 ± 0.00 c
	Compost + SNPs	1.25 ± 0.00 p	1.25 ± 0.00 p	52.8 ± 0.02 d	52.8 ± 0.02 e	1.40 ± 0.00 c	1.50 ± 0.00 c
Soil 3	Control	1.41 ± 0.01 f	1.43 ± 0.01 d	46.7 ± 0.25 n	45.9 ± 0.25 q	1.00 ± 0.00 g	0.90 ± 0.00 i
	Compost	1.31 ± 0.00 k	1.29 ± 0.00 l	50.5 ± 0.02 i	51.3 ± 0.02 i	1.30 ± 0.00 d	1.40 ± 0.00 d
	ES	1.36 ± 0.00 j	1.32 ± 0.00 k	48.7 ± 0.09 j	50.2 ± 0.08 j	1.19 ± 0.00 e	1.39 ± 0.00 d
	SNPs	1.36 ± 0.00 j	1.36 ± 0.00 h	48.7 ± 0.01 j	48.7 ± 0.01 m	1.20 ± 0.00 e	1.30 ± 0.00 e
	Compost + ES	1.27 ± 0.00 n	1.26 ± 0.00 o	52.1 ± 0.02 f	52.4 ± 0.02 f	1.50 ± 0.00 b	1.60 ± 0.00 b
	Compost + SNPs	1.28 ± 0.00 m	1.26 ± 0.00 o	51.7 ± 0.02 g	52.5 ± 0.02 f	1.40 ± 0.00 c	1.50 ± 0.00 c
Soil 4	Control	1.47 ± 0.01 a	1.48 ± 0.01 a	44.4 ± 0.26 s	44.0 ± 0.26 t	0.70 ± 0.00 j	0.60 ± 0.00 j
	Compost	1.45 ± 0.00 c	1.43 ± 0.00 d	45.3 ± 0.03 q	46.0 ± 0.03 q	0.90 ± 0.00 h	1.00 ± 0.00 h
	ES	1.46 ± 0.00 b	1.44 ± 0.00 c	44.9 ± 0.09 r	45.7 ± 0.09 r	0.89 ± 0.00 h	0.99 ± 0.00 h
	SNPs	1.46 ± 0.00 b	1.45 ± 0.00 b	44.9 ± 0.01 r	45.3 ± 0.01 s	0.80 ± 0.00 i	0.90 ± 0.00 i
	Compost + ES	1.42 ± 0.00 e	1.40 ± 0.00 f	46.4 ± 0.02 o	47.1 ± 0.02 o	1.10 ± 0.00 f	1.30 ± 0.00 e
	Compost + SNPs	1.43 ± 0.00 d	1.41 ± 0.00 e	46.0 ± 0.03 p	46.8 ± 0.02 p	1.10 ± 0.00 f	1.20 ± 0.00 f

Means followed by different letters are significant according to the Tukey’s test at *p* ≤ 0.05. Data are means ± SD. *n* = 3.

**Table 4:** Variation in soil nutritional status across different salinity levels—Soil 1 (EC = 3.68 dS/m), Soil 2 (EC = 6.15 dS/m), Soil 3 (EC = 8.34 dS/m), Soil 4 (EC = 12.18 dS/m)—and soil amendments (compost, elemental sulfur (ES), sulfur nanoparticles (SNPs), and their combinations) after growing maize plants (*Zea mays* L., cv Sakha 168) in two consecutive seasons (2022 and 2023).

		Available N (mg/kg)		Available P (mg/kg)		Available K (mg/kg)		Available S (mg/kg)	
		2022	2023	2022	2023	2022	2023	2022	2023
Soil 1	Control	39.8 ± 0.19 de	40.4 ± 0.19 l	4.2 ± 0.02 m	4.3 ± 0.02 m	417 ± 2.0 k	417 ± 2.0 k	15.9 ± 0.07 q	16.8 ± 0.08 p
	Compost	55.3 ± 0.03 b	56.5 ± 0.03 e	5.2 ± 0.00 e	5.3 ± 0.00 f	529 ± 0.3 e	532 ± 0.3 e	19.2 ± 0.01 k	20.3 ± 0.01 k
	ES	51.7 ± 0.09 bc	52.8 ± 0.09 g	3.5 ± 0.01 p	3.5 ± 0.01 o	473 ± 0.8 i	473 ± 0.8 i	22.6 ± 0.04 g	23.5 ± 0.04 g
	SNPs	53.1 ± 0.01 bc	53.8 ± 0.01 f	4.5 ± 0.00 j	4.7 ± 0.00 k	505 ± 0.1 g	506 ± 0.1 g	25.7 ± 0.01 c	26.9 ± 0.01 c
	Compost + ES	67.8 ± 0.02 a	70.2 ± 0.02 b	6.7 ± 0.00 b	6.9 ± 0.00 b	558 ± 0.2 b	567 ± 0.2 b	31.0 ± 0.01 b	31.7 ± 0.01 b
	Compost + SNPs	68.3 ± 0.03 a	70.9 ± 0.03 a	7.1 ± 0.00 a	7.1 ± 0.00 a	567 ± 0.3 a	572 ± 0.3 a	34.2 ± 0.02 a	34.2 ± 0.02 a
Soil 2	Control	40.5 ± 0.19 de	38.8 ± 0.18 m	3.1 ± 0.01 s	3.2 ± 0.01 r	404 ± 1.9 l	407 ± 1.9 l	14.2 ± 0.07 s	14.2 ± 0.07 s
	Compost	47.7 ± 0.02 bcd	47.4 ± 0.02 i	5.1 ± 0.00 h	5.3 ± 0.00 g	522 ± 0.3 f	527 ± 0.3 f	15.4 ± 0.01 r	16.2 ± 0.01 r
	ES	45.1 ± 0.08 cd	46.6 ± 0.08 j	3.3 ± 0.01 r	3.3 ± 0.01 q	466 ± 0.8 j	470 ± 0.8 j	18.8 ± 0.03 l	18.9 ± 0.03 l
	SNPs	46.4 ± 0.01 bcd	47.9 ± 0.01 h	4.4 ± 0.00 k	4.5 ± 0.00 l	499 ± 0.1 h	502 ± 0.1 h	20.1 ± 0.00 j	20.8 ± 0.00 j
	Compost + ES	64.8 ± 0.02 a	66.9 ± 0.02 d	5.3 ± 0.00 d	5.4 ± 0.00 e	539 ± 0.2 d	542 ± 0.2 d	23.2 ± 0.01 f	23.6 ± 0.01 f
	Compost + SNPs	66.2 ± 0.03 a	68.2 ± 0.03 c	5.6 ± 0.00 c	5.9 ± 0.00 d	554 ± 0.3 c	561 ± 0.3 c	25.5 ± 0.01 d	25.8 ± 0.01 d
Soil 3	Control	19.3 ± 0.09 ghi	20.7 ± 0.10 v	3.9 ± 0.02 o	3.0 ± 0.01 t	264 ± 1.2 t	266 ± 1.2 u	13.1 ± 0.06 t	13.3 ± 0.06 u
	Compost	28.3 ± 0.01 fgh	30.2 ± 0.01 q	5.0 ± 0.00 i	5.0 ± 0.00 j	336 ± 0.2 o	339 ± 0.2 o	16.6 ± 0.01 o	16.5 ± 0.01 q
	ES	25.2 ± 0.04 fghi	26.2 ± 0.04 r	4.1 ± 0.01 n	4.2 ± 0.01 n	333 ± 0.6 p	335 ± 0.6 q	21.1 ± 0.04 i	21.0 ± 0.04 i
	SNPs	22.7 ± 0.00 ghi	23.3 ± 0.00 t	3.1 ± 0.00 t	3.2 ± 0.00 r	329 ± 0.1 q	331 ± 0.1 r	17.3 ± 0.00 n	17.9 ± 0.00 n
	Compost + ES	38.6 ± 0.01 de	40.6 ± 0.01 k	5.2 ± 0.00 f	5.3 ± 0.00 h	344 ± 0.1 m	357 ± 0.1 m	24.2 ± 0.01 e	24.4 ± 0.01 e
	Compost + SNPs	33.4 ± 0.02 ef	34.7 ± 0.02 o	5.3 ± 0.00 d	5.9 ± 0.00 c	338 ± 0.2 n	340 ± 0.2 o	22.4 ± 0.01 h	22.9 ± 0.01 h
Soil 4	Control	15.5 ± 0.07 i	17.0 ± 0.08 x	2.1 ± 0.00 w	2.1 ± 0.01 v	262 ± 1.2 u	264 ± 1.2 v	9.9 ± 0.05 w	9.7 ± 0.05 x
	Compost	23.7 ± 0.01 ghi	25.8 ± 0.01 s	3.3 ± 0.01 q	3.4 ± 0.00 p	333 ± 0.2 p	334 ± 0.2 q	11.7 ± 0.01 u	10.3 ± 0.01 w
	ES	21.5 ± 0.04 ghi	22.4 ± 0.04 u	3.1 ± 0.00 u	3.2 ± 0.01 s	300 ± 0.5 r	301 ± 0.5 s	15.4 ± 0.03 r	13.4 ± 0.02 t
	SNPs	19.1 ± 0.00 hi	19.7 ± 0.00 w	2.2 ± 0.01 v	2.2 ± 0.00 u	297 ± 0.1 s	299 ± 0.1 t	10.7 ± 0.00 v	12.8 ± 0.00 v
	Compost + ES	34.7 ± 0.01 ef	36.7 ± 0.01 n	4.3 ± 0.00 l	4.5 ± 0.00 l	335 ± 0.1 o	346 ± 0.1 n	17.5 ± 0.01 m	18.1 ± 0.01 m
	Compost + SNPs	29.1 ± 0.01 fg	30.6 ± 0.01 p	5.1 ± 0.00 g	5.2 ± 0.00 i	339 ± 0.2 n	338 ± 0.2 p	16.2 ± 0.01 p	17.3 ± 0.01 o

Means followed by different letters are significant according to the Tukey’s test at *p* ≤ 0.05. Data are means ± SD. *n* = 3.

**Table 5:** Chemical properties of compost in 2022 and 2023 seasons.

Property	2022	2023
pH (1:10 suspension)	6.82 ± 0.02	6.84 ± 0.01
EC (1:10 suspension) (dS/m)	3.57 ± 0.12	3.45 ± 0.11
Organic matter (%)	38.35 ± 0.23	38.43 ± 0.24
N (%)	1.48 ± 0.01	1.46 ± 0.01
C (%)	32.62 ± 0.21	33.16 ± 0.23
C:N	22.19 ± 0.15	22.87 ± 0.19
P (%)	0.75 ± 0.01	0.74 ± 0.01
K (%)	1.38 ± 0.01	1.27 ± 0.02
Mn (ppm)	297 ± 25	288 ± 19
Fe (ppm)	327 ± 23	334 ± 26
Zn (ppm)	77 ± 1.2	75 ± 1.5

**Table 6:** Mean values for some physical and chemical properties of the experimental soils (0–20 cm) before cultivation during the 2023 and 2024 seasons.

Property	Unit	Soil 1		Soil 2		Soil 3		Soil 4	
		2022	2023	2022	2023	2022	2023	2022	2023
EC (soil paste extract)	dS/m	3.70 ± 0.01	3.67 ± 0.02	6.20 ± 0.02	6.10 ± 0.01	8.39 ± 0.02	8.29 ± 0.01	12.07 ± 0.02	12.30 ± 0.03
pH (1:2.5 suspension)		8.11 ± 0.01	8.12 ± 0.01	8.37 ± 0.02	8.34 ± 0.01	8.66 ± 0.01	8.65 ± 0.02	8.74 ± 0.02	8.72 ± 0.01
SOM	%	1.12 ± 0.01	1.11 ± 0.01	1.12 ± 0.01	1.06 ± 0.01	1.08 ± 0.01	1.05 ± 0.01	0.88 ± 0.01	0.86 ± 0.01
CaCO_3_	%	2.2 ± 0.01	2.2 ± 0.01	2.9 ± 0.02	2.9 ± 0.03	3.2 ± 0.01	3.2 ± 0.02	3.3 ± 0.01	3.2 ± 0.02
SAR		9.6 ± 0.04	9.9 ± 0.05	13.0 ± 0.04	12.0 ± 0.03	16.0 ± 0.04	14.7 ± 0.05	17.6 ± 0.04	17.7 ± 0.02
ESP	%	11.4 ± 0.11	11.7 ± 0.10	15.2 ± 0.11	14.1 ± 0.10	18.2 ± 0.16	16.9 ± 0.12	19.8 ± 0.12	19.9 ± 0.15
Na^+^	meq/L	17.8 ± 0.15	17.1 ± 0.11	38.3 ± 0.25	30.3 ± 0.15	48.5 ± 0.25	45.5 ± 0.35	66.1 ± 0.25	63.1 ± 0.25
Ca^++^	meq/L	6.4 ± 0.09	5.5 ± 0.07	11.9 ± 0.05	11.0 ± 0.09	15.2 ± 0.19	14.0 ± 0.09	20.2 ± 0.08	19.4 ± 0.19
Mg^++^	meq/L	4.2 ± 0.02	3.6 ± 0.01	7.7 ± 0.02	7.2 ± 0.03	8.7 ± 0.02	8.5 ± 0.04	12.1 ± 0.02	11.8 ± 0.05
K^+^	meq/L	0.42 ± 0.01	0.36 ± 0.01	0.79 ± 0.02	0.72 ± 0.01	0.99 ± 0.03	0.52 ± 0.01	0.74 ± 0.02	0.72 ± 0.01
CO_3_^−−^	meq/L	0 ± 0.0	0 ± 0.0	0 ± 0.0	0 ± 0.0	0 ± 0.0	0 ± 0.0	0 ± 0.0	0 ± 0.0
HCO_3_^−^	meq/L	1.2 ± 0.01	1.4 ± 0.01	2.6 ± 0.01	3.1 ± 0.01	4.5 ± 0.02	5.0 ± 0.01	6.0 ± 0.01	6.7 ± 0.02
Cl^−^	meq/L	14.3 ± 0.13	13.7 ± 0.13	30.7 ± 0.14	24.3 ± 0.17	41.3 ± 0.19	31.8 ± 0.18	46.2 ± 0.21	44.1 ± 0.23
SO_4_^−−^	meq/L	13.3 ± 0.14	11.4 ± 0.15	25.4 ± 0.13	21.8 ± 0.13	27.6 ± 0.19	31.7 ± 0.14	46.9 ± 0.19	44.3 ± 0.13
N	mg/kg	39.7 ± 1.11	40.3 ± 1.31	40.4 ± 1.12	38.7 ± 1.11	19.2 ± 1.02	20.7 ± 0.11	15.5 ± 0.08	17.0 ± 0.09
P	mg/kg	4.23 ± 0.02	4.32 ± 0.02	3.12 ± 0.01	3.18 ± 0.01	3.93 ± 0.02	2.95 ± 0.03	2.12 ± 0.02	2.13 ± 0.01
K	mg/kg	416 ± 33	416 ± 35	403 ± 34	406 ± 37	263 ± 21	265 ± 20	261 ± 19	263 ± 21
Sulfur	%	15.9 ± 0.13	16.7 ± 0.16	14.2 ± 0.15	14.2 ± 0.17	13.1 ± 0.15	13.2 ± 0.13	9.9 ± 0.10	9.6 ± 0.11
Field capacity (FC)	%	42.9 ± 1.05	43.8 ± 1.11	40.7 ± 1.11	40.7 ± 1.13	40.6 ± 1.22	40.5 ± 1.12	39.8 ± 1.22	40.2 ± 1.12
Wilting point (PWP)	%	22.1 ± 0.56	22.3 ± 0.54	21.3 ± 0.55	21.5 ± 0.44	21.5 ± 0.43	21.7 ± 0.42	20.2 ± 0.41	21.2 ± 0.36
Soil bulk density	kg/m^3^	1.37 ± 0.02	1.38 ± 0.01	1.39 ± 0.02	1.38 ± 0.01	1.41 ± 0.01	1.43 ± 0.02	1.47 ± 0.01	1.48 ± 0.02
Total porosity	%	48.3 ± 1.12	47.9 ± 1.15	47.5 ± 1.12	47.9 ± 1.16	46.8 ± 1.18	46.0 ± 1.17	44.5 ± 1.11	44.2 ± 1.10
Infiltration rate	(cm/h)	1.2 ± 0.01	1.3 ± 0.01	1.1 ± 0.01	1.1 ± 0.01	1.0 ± 0.01	0.9 ± 0.01	0.7 ± 0.01	0.6 ± 0.01
sand	%	15.95 ± 0.36	15.87 ± 0.33	14.89 ± 0.36	15.07 ± 0.34	14.75 ± 0.36	14.62 ± 0.32	13.98 ± 0.31	14.06 ± 0.30
silt	%	32.25 ± 0.55	32.57 ± 0.56	32.87 ± 0.54	32.56 ± 0.43	32.92 ± 0.42	32.91 ± 0.41	33.45 ± 0.41	32.90 ± 0.36
clay	%	51.80 ± 1.05	51.56 ± 1.11	52.23 ± 1.13	52.37 ± 1.11	52.33 ± 1.22	52.47 ± 1.22	52.57 ± 1.12	53.04 ± 1.22
Texture class		clay	clay	clay	clay	clay	clay	clay	clay

**Table 7:** Properties of irrigation water during the two growing seasons.

Property	2022	2023
pH	7.58 ± 0.01	7.96 ± 0.01
EC (dS/m)	0.75 ± 0.03	0.78 ± 0.02
SAR	4.58 ± 0.03	4.68 ± 0.01
Na^+^ (meq/L)	5.09 ± 0.03	5.31 ± 0.02
Ca^++^ (meq/L)	1.57 ± 0.02	1.63 ± 0.01
Mg^++^ (meq/L)	0.90 ± 0.01	0.94 ± 0.01
K^+^ (meq/L)	0.06 ± 0.001	0.06 ± 0.001
CO_3_^−−^ (meq/L)	0 ± 0.0	0 ± 0.0
HCO_3_^−^ (meq/L)	1.5 ± 0.01	1.7 ± 0.002
Cl^−^ (meq/L)	3.56 ± 0.03	4.40 ± 0.03
SO_4_^−−^ (meq/L)	2.56 ± 0.02	1.85 ± 0.01

## Data Availability

Data will be made available upon the request.
